# Relationship between benzene concentration, MDA levels and kidney function in car painting workshops in Surabaya: A cross-sectional observational study

**DOI:** 10.12688/f1000research.140287.1

**Published:** 2023-10-30

**Authors:** Abdul Rohim Tualeka, Mohd Yusmaidie Aziz, Velu Perumal, Tamilanban Thamaraikani, Roslan Rosnon, Salsabila Novianti, Pudji Rahmawati, Ahsan Ahsan

**Affiliations:** 1 Department of Occupational Health and Safety, Faculty of Public Health, Airlangga University, Surabaya, East Java, 60115, Indonesia; 2 Department of Toxicology, Advanced Medical & Dental Institute, Universiti Sains Malaysia, Bertam Kepala Batas, Penang, 13200, Malaysia; 3 Jabatan Rekabentuk Perindustrian Fakulti Rekabentuk dan Senibina, Universiti Putra Malaysia, Serdang, Selangor, 43400, Malaysia; 4 Department of Pharmacology, SRM College of Pharmacy,, SRM Institute of Science and Technology (Deemed to be University), Kattankulathur, Tamil Nadu, 603203, India; 5 Department of Social & Development Sciences, Faculty of Human Ecology, Universiti Putra Malaysia, Serdang, Selangor, 43400, Malaysia; 6 Department of Environmental Health, Faculty of Public Health,, Airlangga University, Surabaya, East Java, 60115, Indonesia; 7 Department of Development of Islamic Society, Sunan Ampel State Islamic University Surabaya, Surabaya, East Java, Indonesia; 8 Faculty of Nursing, Brawijaya University, Malang, East Java, Indonesia

**Keywords:** Benzene, malondialdehyde, kidney function, car painting workshops, safe work

## Abstract

**Background:**

Car painting workers are at risk because of the use of solvents containing benzene as the main ingredient in the car painting process. One of the clinical effects of systemic benzene is kidney disorders. Therefore, the purpose of this study is to analyze the relationship between benzene and Risk Quotient (RQ) benzene concentrations with Malondialdehyde (MDA), Blood Urea Nitrogen (BUN), and creatinine levels in workers exposed to benzene in car painting workshops in Surabaya.

**Methods:**

This is an observational, cross-sectional study conducted at two car painting workshops in Surabaya that use benzene as a solvent in their production process, namely in industries in Kalijudan and Jemursari. The research sample was taken using the accidental sampling method and as many as 30 respondents were involved in this study. The variables studied included benzene concentration, RQ benzene, MDA levels, and kidney function (BUN and creatinine levels). Analysis of the data used is a descriptive and bivariate analysis using the Pearson correlation test.

**Results:**

There was no significant relationship between concentrations, RQ benzene, and MDA levels in workers in painting in Surabaya (p> 0.05). There was no significant relationship between benzene concentration, BUN levels, and creatinine levels in paint workers in Surabaya (p> 0.05). There was no significant relationship between benzene RQ and BUN and creatinine levels in paint workers in Surabaya (p> 0.05).

**Conclusions:**

The results of this study indicate that the effects of benzene do not lead to impaired kidney function. The benzene RQ variable in this study did not become a determining factor in BUN and creatinine levels in workers.

## Introduction

Benzene is a carcinogenic unsaturated closed-chain aromatic hydrocarbon compound ( ATSDR, 2007). Benzene has been known as a good organic solvent for various processes in the industry such as the rubber industry, shoes, paint solvents, components in motor fuels, components in detergents, pesticides, and pharmaceutical manufacturing ( Paustenbach et al., 1992; Wijaya, 1993). One informal sector that is often exposed to benzene is the car paint shop. The car painting work area is one of the areas that requires attention due to its increasing number with a large risk of occupational diseases. The car painting process uses solvents containing benzene as the main ingredient in the work process which can have a detrimental effect on health. These materials enter the body through absorption with more presentation through inhalation due to exposure to steam in the process of spray painting ( Coresh et al., 2007).

Continuous benzene exposure can cause health effects. The body is continually exposed to benzene which causes symptoms and signs of chronic poisoning such as headaches, dizziness, nausea to vomiting, and slow-in-pale reactions due to anemia which is often accompanied by bleeding under the skin and mucosa. The clinical effects of benzene systemically cause cardiovascular, respiratory, neurological, gastrointestinal, liver, kidney, endocrine and reproductive systems, dermatology, local effects, hematological, immunological, metabolic, and allergic reactions ( ATSDR, 2007; Cronkite et al., 1989; McHale et al., 2012; Tunsaringkarn et al., 2013).

In 2007 the ( American Conference of Governmental Industrial Hygienists (ACGIH), 2007) issued a benzene chemical threshold of 0.5 ppm and since 1997 benzene has been confirmed to have carcinogenic properties in humans (Al = confirmed human carcinogen). The National Institute for Occupational Health and Safety (NIOSH) in 2010 set a recommended exposure limit or REL (Recommended Exposure Limit) of 0.1 ppm for 8 working hours ( NIOSH, 2010). The threshold value of chemical factors at work according to Minister of Manpower and Transmigration number 13 in 2011 is 0.5 ppm. The benzene exposure pathway enters the human body in three ways, namely absorption through the skin, inhalation, and ingestion. Inhalation is a very important route to consider because benzene has volatile properties ( ATSDR, 2007).

Benzene which enters the body oxidizes to proteins, lipids and produces Malondialdehyde (MDA). An increase in MDA levels is a sign of an increase in free radicals in the blood. Increased MDA levels even become a benchmark to determine the risk of cancer that will occur in workers exposed to benzene. Exposure to benzene in high content causes narcotic effects and irritation to the eyes and airways ( Ho et al., 2006). Long-term exposure to low content can result in bone marrow suppression and can be associated with leukemia events or other blood cell disorders. The population of workers who work in the car painting industry or use benzene can be exposed to the highest exposure levels. For this reason, special attention needs to be paid to workers for occupational safety and health.

Exposure to benzene and alkyl benzene has been linked to kidney and liver injury and kidney cancer ( Brautbar et al., 2006; Henderson, 2001). Other research conducted in Indonesia also stated that as many as 256 child workers in the Cibaduyut Bandung slipper and shoe industry were listed as being threatened by various types of diseases such as liver and/or kidney damage and even leukemia ( ILO, 2004). That is due to bad habits and an unhealthy work environment so workers in the Cibaduyut Bandung sandal and shoe industry inhale and ingest benzene compounds contained in the glue they use to make sandals.

Examination of creatinine level in the blood is one of the parameters used to assess kidney function, because the concentration in plasma and its excretion in urine in 24 hours is relatively constant ( Soedaman, 1995). This serum creatinine reflects the most sensitive kidney damage because it is produced constantly by the body ( Lewis et al., 2014). In addition, high Blood Urea Nitrogen (BUN) levels have been associated with adverse kidney effects suggesting that BUN is a useful marker for predicting the development of kidney disease ( Seki et al., 2019). Therefore, the purpose of this study is to analyze the relationship between benzene concentration and RQ benzene with MDA, BUN, and creatinine levels in workers exposed to benzene in a car painting workshop in Surabaya.

## Methods

This is an observational, cross-sectional study conducted at 2 car painting workshops in Surabaya that uses benzene as a solvent in its production process, namely in Kalijudan and Jemursari. The population in this study were all 90 workers exposed to benzene in two car painting workshops in Surabaya aged between 20-65 years in 2019. The research sample was taken using accidental sampling method involving 30 respondents. The variables studied were benzene concentration, RQ benzene, MDA levels and kidney function (BUN and creatinine levels). Analysis of the data used is a descriptive and bivariate analysis using Pearson correlation test.

In this study, the variables under investigation encompassed benzene concentration, RQ benzene (benzene metabolite), levels of malondialdehyde (MDA) as a marker of oxidative stress, and indicators of kidney function such as blood urea nitrogen (BUN) and creatinine levels. Benzene concentration and RQ benzene were considered exposures, representing the extent of exposure to benzene and its metabolic byproduct, respectively. MDA levels were examined as an outcome, reflecting oxidative stress status. Kidney function parameters (BUN and creatinine levels) were outcomes, indicating potential renal effects. To account for potential influences on the relationships, age and duration of exposure were treated as potential confounders, while smoking status was considered an effect modifier due to its potential interaction with benzene exposure.

### Data measurements and bias

Benzene concentration and RQ benzene data were collected through air sampling in the workplace, utilizing gas chromatography as the assessment method. MDA levels, indicative of oxidative stress, were measured using spectrophotometric assays on blood samples. Kidney function markers, BUN, and creatinine levels, were assessed through blood tests conducted at a clinical laboratory. Efforts were made to address participant selection bias through the accidental sampling method. While this method might introduce some bias due to its non-random nature, its pragmatic approach allowed for data collection from the available workforce, considering practical constraints. The sample size, comprising 30 respondents, was determined based on available resources while aiming to capture a representative subset of the population.

The study employed a comparative approach between the two workshops to explore potential differences in variables of interest and their interactions. This approach facilitated a more nuanced understanding of the relationship between benzene exposure, MDA levels, and kidney function within the specific context of the car painting workshops in Surabaya.

## Results

### Characteristics of Respondents Exposed to Benzene at a Car Painting Workshop in Surabaya

Respondent characteristics include age, sex, level of education, and work area. Table 1 presents the distribution of characteristics of workers exposed to Benzene in a car painting workshop in Surabaya.

**Table 1.** Distribution of Characteristics of Workers Exposed to Benzene at Car Painting Workshops in Surabaya.

Characteristics of Respondents	Frequency	Percentage
**Age**		
20-25	8	29.6
26-35	3	11.2
36-45	9	33.3
46-55	6	22.2
56-65	1	3.7
**Sex**		
Male	26	96.3
Female	1	3.7
**Level of Education**		
Primary	3	11.1
Junior High	11	40.8
Senior High	12	44.4
University	1	3.7
**Working Area**		
Kalijudan	17	63
Jemursari	10	37

Source: primary data.

Most (33.3%) industrial workers aged 36-45 years and the majority (96.3%) was male with the highest level of education being SMA/SMK (44.4%). Most (63%) workers work in the Kalijudan area.

### Benzene concentration

Based on Table 2 of 27 respondents there were 21 respondents (77.8%) with benzene concentrations above the Threshold Value (> 0.5 ppm) and 6 respondents (22.2%) with benzene concentrations below the Threshold Value (≤ 0.5 ppm).

**Table 2.** Distribution of Benzene Concentration to Workers Exposed to Benzene at Car Painting Workshops in Surabaya.

Benzene Concentration	Total	
	N	%
**>0,05 ppm**	21	77.8
**≤0,05 ppm**	6	22.2
**Total**	27	100

Source: primary data.

### Benzene RQ

Health risk characteristics are stated as Risk Quotient (RQ, Risk Level), shown in Table 3 and are calculated by dividing the intake or intake (Ink) by reference (RfC). The calculation results of Risk Quotients (RQ) can indicate the level of health risks of workers due to exposure to benzene in the work environment. If the RQ value is more than or equal to 1 (RQ> 1) then workers exposed to benzene have health risks due to benzene exposure. If the RQ value is less than 1 (RQ <1), then workers exposed to benzene are safe from health risks due to benzene exposure. Based on the RQ calculation in Table 4, the majority of workers (92.6%) have RQ≥1 values for benzene exposure, which means the majority of them have health risk impacts due to benzene exposure.

**Table 3.** Calculation of RQ on Benzene Workers on Workers Exposed to Benzene at Car Painting Workshops in Surabaya.

No	Benzene Concentration (ppm)	Weight (kg)	tE (hours)	fE (days)	Dt (years)	Breathing Rate (m ^3^/hour)	RFC Benzene	Benzene dose Intake	Benzene RQ
1	0.6768	63	7	312	25	0.627442	0.0003128	0.107155	146.787
2	1.4933	63	7	312	15	0.627442	0.000313	0.141856	194.235
3	1.4933	55.5	9	312	25	0.599451	0.000313	0.329663	451.388
4	0.6768	54	7	312	1.5	0.593401	0.000313	0.007093	9.71323
5	1.4933	49	8	312	2	0.571944	0.000313	0.025334	34.6883
6	1.4933	80	7	312	2	0.680198	0.000313	0.016147	22.1095
7	0.1678	48	7	312	10	0.56739	0.000313	0.012612	17.2699
8	0.1678	63	7	312	11	0.627442	0.000313	0.011689	16.0057
9	0.1678	66	9	312	8	0.637715	0.000313	0.010604	14.5200
10	0.6768	43	8	312	5	0.543098	0.000313	0.031060	42.5295
11	0.6768	54	8	312	3	0.593401	0.000313	0.016214	22.2016
12	0.6768	62	9	312	4	0.623909	0.000313	0.022272	30.4966
13	1.4933	74	11	312	12	0.662981	0.000313	0.160424	219.659
14	1.4933	65	7	312	10	0.634344	0.000313	0.092669	126.886
15	1.4933	72	7	312	20	0.65693	0.000313	0.173277	237.258
16	1.4933	50	7	312	0.08	0.576405	0.000313	0.000908	1.24405
17	1.4933	55	8	312	12	0.597453	0.000313	0.141462	193.695
18	1.3891	70	7	312	0.5	0.650709366	0.000307925	0.00410555	1.3891
19	1.5328	70	7	312	2	0.650709366	0.000307925	0.01812105	1.5328
20	1.3891	45	7	312	1	0.553137966	0.000307925	0.01085759	14.6705
21	14.6705	70	7	312	0.83	0.650709366	0.000307925	0.07197656	1.3891
22	1.3891	55	7	312	18	0.597452745	0.000307925	0.17271336	1.3891
23	1.3891	50	7	312	0.83	0.57640508	0.000307925	0.00845178	1.3891
24	1.3891	85	9	312	10	0.693585486	0.000307925	0.09266977	1.3891
25	1.3891	52	7	312	3	0.585066321	0.000307925	0.02981505	1.3891
26	1.3891	50	7	312	5	0.57640508	0.000307925	0.05091437	0.9282
27	1.3891	47	7	312	15	0.562740929	0.000307925	0.15864065	0.0414
avg	1.653	59.6	7.6	312	8.2	0.611773	0.000311	0.071063	66.8960

Source: primary data.

**Table 4.** Distribution of Benzene RQ Frequency to Workers Exposed to Benzene at Car Painting Workshops in Surabaya.

RQ	Total	
	N	%
**Unsafe (≥1)**	25	92.6
**Safe ( <1)**	2	7.4
**Total**	30	100%

Source: primary data.

### Relationship between Benzene concentration and MDA

Based on the test results in Table 5 there is no significant relationship between Benzene concentration and MDA levels in workers exposed to benzene in a car painting workshop in Surabaya with a P value> 0.05.

**Table 5.** Statistical Test Results between Benzene and MDA Concentrations.

Variables	*P*-Value	Correlation coefficient	N
Benzene concentration	0.179	-0.266	30
MDA			

Source: primary data.

### Relationship between RQ Benzene and MDA

Based on the test results in Table 6 there is no relationship between RQ Benzene and MDA levels of workers exposed to benzene in a car painting workshop in Surabaya (P> 0.05).

**Table 6.** Statistical Test Results between RQ Benzene and MDA.

Variables	*P*-Value	Correlation coefficient	N
Benzene RQ	0.597	0.106	30
MDA			

Source: primary data.

### Relationship between Benzene Concentration and Kidney Function

Based on the test results in Table 7 there was no significant relationship between Benzene concentrations, BUN levels and creatinine exposure of workers exposed to benzene in car painting workshops in Surabaya (P> 0.05).

**Table 7.** Statistical Test Results Between Benzene Concentration and Kidney Function.

Variables	*P*-Value	Correlation coefficient	N
Benzene concentration	0.238	-0.235	30
BUN level			
Benzene concentration	0.790	0.054	30
Creatinine levels			

Source: primary data.

### Relationship between RQ Benzene and Kidney Function

Based on the test results in Table 8 there was no significant relationship between Benzene concentrations, BUN levels, and creatinine exposure of workers exposed to benzene at a car painting workshop in Surabaya (P> 0.05).

**Table 8.** Statistical Test Results Between Benzene Concentration and Kidney Function.

Variables	*P*-Value	Correlation coefficient	N
Benzene RQ	0.537	0.124	30
BUN level			
Benzene RQ	0.397	-0.170	30
Creatinine levels			

Source: primary data.

## Discussion

The results showed that there was no significant relationship between concentration, RQ benzene, and MDA levels in workers in a car painting workshop in Surabaya (p> 0.05). This is in line with research conducted on workers in shoe factories that benzene concentrations do not have a significant relationship with MDA levels ( Tualeka et al., 2019). However, according to research conducted by ( Odewabi et al., 2014) in Nigeria, exposure to free radicals especially benzene in gas station workers can increase MDA levels in workers. Research by ( Suparno et al., 2018) also stated that high plasma malondialdehyde (MDA) levels are markers of oxidative stress that will cause DNA and RNA disturbances. Previous research suggests that oxidative stress might be related to pathogenesis and the dev1elopment of kidney disease, where it is suspected that malondialdehyde might play an important role in the pathogenesis of glomerulosclerosis ( Kuo et al., 2005). In other studies, oxidative stress has progressively increased and is associated with the degree of kidney dysfunction in patients with chronic kidney failure ( Dounousi et al., 2006; Terawaki et al., 2004).

There was no significant relationship between benzene exposure, BUN levels and creatinine in painting workers in Surabaya (p> 0.05). Research conducted by ( D’Andrea & Reddy, 2018) in children showed no significant differences in serum creatinine levels between groups exposed to benzene and those not exposed. Although BUN levels were found to be significantly reduced in groups exposed to benzene compared with unexposed group (P = 0.001). Although studies related to the effects of benzene exposure specifically on kidney function (creatinine and BUN) are limited, previous studies related to the exposure of organic solvents to kidney function have been conducted to support this study. Research conducted by ( Elfar et al., 1998) found no statistically significant differences between the groups exposed to organic solvents and the control group regarding kidney function and there was no significant relationship between the two and the length of exposure to organic solvents. This opinion is also strengthened by research conducted by ( Kaukiainen et al., 2004) who found a negative relationship between serum creatinine levels and exposure to organic solvents.

( Hoek et al., 2003) did not find any effect from exposure to organic solvents on effects to the kidneys. The lack of an association between kidney effects and the intensity or duration of exposure can be associated with individual vulnerability. Vulnerability to benzene can vary due to its effects which arise, in part, from genetic variations in metabolism, DNA repair, genome stability, and immune function ( D’Andrea & Reddy, 2018). In the present study, the effects of benzene have not led to impaired kidney function, yet limited to acute exposure. In addition, the presence of toluene exposure inhaled by labor (measured at the same time as the measurement of benzene exposure using the OVM method) has antagonistic properties against benzene toxicity. According to ( Inoue et al., 1988) workers exposed to a combination of benzene and toluene will experience decreased levels of phenol in the urine compared to those exposed to benzene or toluene separately. Therefore, further research can find out whether there is an antagonistic effect between benzene and toluene on creatinine and BUN levels.

There was no significant relationship between benzene RQ, BUN levels and creatinine levels in paint workers in Surabaya (p> 0.05). RQ calculation is calculated by dividing the intake or intake (ink) by reference (RfC). Therefore, one factor that influences the value of RQ is the amount of benzene intake. Based on the theory of ( Louvar & Louvar, 1998) in determining the assessment of exposure (exposure assessment) regarding the amount of chemical intake received by individuals, the exposure time factor, duration of exposure, body weight and frequency of exposure have a significant contribution in determining the intake of xenobiotic material intake at body to cause health effects. Other factors such as duration of exposure, time of exposure, frequency of exposure, nutritional status, etc. can contribute in the event that there is no effect of the benzene RQ variable on kidney function. In this study, benzene intake in workers was relatively small and the work period of the worker was also not long at 8.2 years (<10 years). In sum, the benzene RQ variable in this study was not a determining factor in its effect on the results of BUN and creatinine levels.

### Limitations

Cross-sectional design restricts the ability to establish causal relationships between variables, which means that it captures data at a single point in time. Longitudinal studies would provide more robust evidence of the relationship between these variables. The study is conducted in only two car painting workshops in Surabaya, potentially limiting generalizability to other settings. The utilization of the accidental sampling method might introduce selection bias, as workers with greater awareness of health risks might be more inclined to participate, affecting the external validity of findings. Furthermore, age range selection (20-65 years) might lead to varying susceptibility levels and differences in exposure duration, potentially influencing the direction and magnitude of associations observed. The limited sample size (30 respondents) might hinder the ability to detect small but significant effects, impacting the study's statistical power. As the study relies on self-reported data for certain variables and uses laboratory assessments for others, measurement bias and misclassification could occur. Despite these limitations, the study's findings contribute to the existing understanding of the complex interplay between benzene exposure, oxidative stress, and kidney function in car painting workshops.

## Conclusion

In this study, the majority of respondents (77.8%) were exposed to benzene concentrations above the Threshold Value (>0.5 ppm). The majority of workers (92.6%) had RQ≥1 against benzene exposure, which means the majority of workers were affected by benzene exposure. There was no significant relationship between benzene concentration and RQ on MDA level in workers (p>0.05). There was no significant relationship between benzene concentration, BUN level, and creatinine level in workers (p>0.05). There was no significant relationship between benzene RQ, BUN level, and creatinine level in workers (p>0.05).

### Ethical statement

Ethical approval was obtained from the Universitas Airlangga, Faculty of Dental Medicine ethics committee (605/HRECC.FODM/IX/2019).

### Informed consent

The objectives and protocols of the study were explained to the participants and obtained written informed consent from each subject before participation in the study.

## Data Availability

All data underlying the results are available as part of the article and no additional source data are required.
