# Supplementary material for: Relationship between benzene concentration, MDA levels and kidney function in car painting workshops in Surabaya: A cross-sectional observational study
Source: F1000Res. 2025 Sep 5;12:1419. Originally published 2023 Oct 30. [Version 3] doi: 10.12688/f1000research.140287.3 (PMC12457878; doi:10.12688/f1000research.140287.3)
Supplement: Peer review history

# Reviewer report: Reviewer response for version 3

**DOI:** 10.5256/f1000research.186720.r412476

**Published:** 2025-09-23

**Authors:** Chan Pattama Polyong

**Affiliations:** 1 Bansomdejchaopraya Rajabhat University, Bangkok, Thailand

**Reasons for Rejection**

**Major Concerns**

**Outdated or Misapplied Standards**           

 -The manuscript relies on outdated occupational exposure limits (e.g., ACGIH TLV of 0.5 ppm), whereas the most recent TLV (2025) is 0.02 ppm. Mixing different reference values (OSHA PEL, NIOSH REL, ACGIH TLV, national regulations) without justification undermines the validity of the conclusions.

  -Reference to ATSDR documents is inconsistent (2007 vs. 2025), and the source and value of the reference concentration (RfC) for risk calculations are not clearly stated.

**Misdefinition and Misuse of the “Risk Quotient (RQ)”**           

 -The paper incorrectly defines RQ as a metabolite of benzene measured by gas chromatography. In toxicology, RQ (or Hazard Quotient) is the ratio of exposure/intake to a reference concentration or dose, not a laboratory measurement.

 -Without a clear definition, formula, and source of RfC, the reported RQ values are not meaningful or interpretable.

**Weak Study Design and Sampling**           

 -Cross-sectional design with accidental sampling (n=30) from only two workshops has low external validity and high selection bias.

 -Inconsistency in sample size reporting (27 vs. 30 participants) is problematic.

 -“Smoking” is cited as an effect modifier, yet no stratified or interaction analysis is presented.

**Exposure Assessment and Quality Control**           

 -Details about air sampling are insufficient: it is unclear whether personal or area samples were collected, for how long, and according to which standardized method (e.g., NIOSH 1501).

 -No information is provided on LOD/LOQ, calibration, blanks, or QA/QC procedures.

 -Mention of OVM in the discussion is inconsistent with the methods section.

**Non-Specific Biomarkers**

 -Malondialdehyde (MDA) is not a specific biomarker of benzene exposure and is prone to methodological interferences.

   -More specific biomarkers such as S-phenylmercapturic acid (SPMA) or trans,trans-muconic acid (tt-MA) should have been used.

  -Kidney function was assessed only with BUN and creatinine, which are insensitive to early renal injury. More sensitive markers (e.g., KIM-1, NGAL, NAG, β2-microglobulin, eGFR, albuminuria) are recommended.

**Inadequate Statistical Analysis and Reporting**            -Results are presented only as p-values without measures of central tendency, variability, effect size, or confidence intervals.

 -No multivariable analysis to adjust for confounding factors (age, duration of exposure, smoking, co-exposures such as toluene) was performed.

 -Methods for statistical testing are not specified.

**Over-Interpretation and Internal Inconsistency**           

 -The paper concludes that workers are “affected” by benzene exposure (based on RQ≥1) despite finding no association with MDA, BUN, or creatinine.

  -Antagonistic effects of toluene are discussed without having measured toluene exposure.

 -The introduction broadly discusses systemic toxicity but the study only measures oxidative stress and crude renal endpoints.

**Ethical and Occupational Health Issues**

 -No mention of ethical approval, informed consent, or follow-up for at-risk workers.

 - No recommendations for occupational control measures (e.g., substitution, ventilation, respiratory protection), which are critical when reporting high exposure levels.

**Minor but Important Issues**

**-Language and Terminology**: The manuscript contains repetitive and confusing phrasing (e.g., “benzene RQ and benzene concentration…”).

**-Tables and Figures**: Tables are incomplete, lack units, and contain inconsistencies (e.g., decimal separators, missing totals).

**- References**: Citations are inconsistent in style and some are outdated. Authors should update references to the latest authoritative sources (e.g., ACGIH TLV 2025, ATSDR 2025).

**- Formatting**: There are errors in decimal notation (commas vs. points), which can lead to misinterpretation of values.

 Even though this study has major methodological limitations and requires substantial revision, it is clear that the authors have made an important effort to address a relevant occupational health issue. With a stronger study design, updated exposure assessment, and more specific biomarkers, future research from this team could make a meaningful contribution to the field. I encourage the authors to continue developing their work in this area.

Is the work clearly and accurately presented and does it cite the current literature?

Partly

If applicable, is the statistical analysis and its interpretation appropriate?

No

Are all the source data underlying the results available to ensure full reproducibility?

No

Is the study design appropriate and is the work technically sound?

No

Are the conclusions drawn adequately supported by the results?

Partly

Are sufficient details of methods and analysis provided to allow replication by others?

No

Reviewer Expertise:

Occupational Health, Toxicology

I confirm that I have read this submission and believe that I have an appropriate level of expertise to state that I do not consider it to be of an acceptable scientific standard, for reasons outlined above.

# Reviewer report: Reviewer response for version 3

**DOI:** 10.5256/f1000research.186720.r411883

**Published:** 2025-09-23

**Authors:** Reny Indrayani

**Affiliations:** 1 University of Jember, Jember Regency, Indonesia

The author has responded to most of the comments.

Is the work clearly and accurately presented and does it cite the current literature?

No

If applicable, is the statistical analysis and its interpretation appropriate?

Partly

Are all the source data underlying the results available to ensure full reproducibility?

Partly

Is the study design appropriate and is the work technically sound?

Partly

Are the conclusions drawn adequately supported by the results?

Partly

Are sufficient details of methods and analysis provided to allow replication by others?

No

Reviewer Expertise:

Occupational Safety and Health; Industrial Hygiene

I confirm that I have read this submission and believe that I have an appropriate level of expertise to confirm that it is of an acceptable scientific standard.

# Reviewer report: Reviewer response for version 2

**DOI:** 10.5256/f1000research.167506.r388556

**Published:** 2025-06-03

**Authors:** Reny Indrayani

**Affiliations:** 1 University of Jember, Jember Regency, Indonesia

1. **Literature:**
  (1) The cited literature is not sufficiently up-to-date (e.g., Inoue et al., 1988).
  (2) Fewer than 30 references are used, and only about 60% of them are derived from scientific journals. Among those, only approximately 35% were published between 2013 and 2023.
  (3) Some references are also not traceable (e.g., Elfar et al., 1998).
**Suggestion:** Increase the number of references, particularly from scientific journals, and prioritize using recent sources (preferably from the last 10 years) to enrich the discussion.
2. **Methods:**
  (1) The statistical analysis used in the study is not described (e.g., what types of tests were applied?).
  (2) The use of accidental sampling is not appropriate for an analytical observational study; random sampling should be used instead.
  (3) The formula for calculating benzene dose intake is not provided.
3. **Results:**
  There is no table presenting the measurement results of MDA, BUN, and creatinine levels.
4. **Discussion:**
  (1) There is no scientific explanation regarding the discrepancies between certain findings and existing theories (e.g., between RQ and MDA). The authors mostly cite previous studies whose results are in agreement with existing theories. Even when prior studies support the current findings, the potential reasons for any observed inconsistencies with established theories remain unexplained.
  (2) The authors briefly mention Cytochrome P450 enzymes (CYP450) as a possible explanation for anomalous findings, but the relevance needs to be clarified. Since CYP450 was not measured in this study, it may be better suggested for future research.
5. **Conclusion:**
  The last two sentences should be combined into one for better clarity and flow.
6. **Other Remarks:**
  a. In the abstract: “Car painting workers are at risk because…” – the type of risk is not specified and should be clarified.
  b. In the introduction:
  (1) The statement “Minister of Manpower and Transmigration number 13 in 2011 is 0.5 ppm” refers to a regulation that is no longer valid, as it has been replaced by a newer regulation issued in 2018. It should also be clarified that this regulation applies in Indonesia.
  (2) The sentence “Benzene which enters the body oxidizes to proteins, lipids and produces Malondialdehyde (MDA)” is incorrect. Benzene is not oxidized into proteins or lipids; rather, its metabolism increases reactive oxygen species (ROS), which act as oxidants.
  (3) There is no explanation provided about the Respiratory Quotient (RQ) or the purpose of calculating it, although it is suddenly included in the study objectives.

Is the work clearly and accurately presented and does it cite the current literature?

No

If applicable, is the statistical analysis and its interpretation appropriate?

Partly

Are all the source data underlying the results available to ensure full reproducibility?

Partly

Is the study design appropriate and is the work technically sound?

Partly

Are the conclusions drawn adequately supported by the results?

Partly

Are sufficient details of methods and analysis provided to allow replication by others?

No

Reviewer Expertise:

Occupational Safety and Health; Industrial Hygiene

I confirm that I have read this submission and believe that I have an appropriate level of expertise to confirm that it is of an acceptable scientific standard, however I have significant reservations, as outlined above.

# Reviewer report: Reviewer response for version 2

**DOI:** 10.5256/f1000research.167506.r343487

**Published:** 2024-12-16

**Authors:** Giulia Barbieri

**Affiliations:** 1 University of Verona, Eurac Research, Verona, Italy

This study examined the effects of benzene exposure on oxidative stress (MDA) and kidney function (BUN, creatinine) in 30 car painting workers in Surabaya. No significant relationships were found between benzene levels, oxidative stress, or kidney function markers (p > 0.05), suggesting that benzene exposure did not impair kidney function or cause oxidative stress at the levels studied.

 The most problematic aspect of the work is the absence of description of the methods employed for the statistical analysis, which are just briefly mentioned in the abstract. Smoking status is mentioned as effect modifier, but then univariate analysis are performed without taking it into account.

 Data to replicate the above-mentioned analyses are not sufficient.

 The evidence from results is very weak (univariate analyses, small samplesize, etc)  and prevents the causal interpretation of associations.

 Supporting literature is not accurate, some links are broken (e.g. NIOSH), others are misplaced (e.g. Coresh et al), reporting imprecise evidences (e.g. D'Andrea et al), or even not existing (e.g. Elfar et al).

Is the work clearly and accurately presented and does it cite the current literature?

No

If applicable, is the statistical analysis and its interpretation appropriate?

Partly

Are all the source data underlying the results available to ensure full reproducibility?

Partly

Is the study design appropriate and is the work technically sound?

Partly

Are the conclusions drawn adequately supported by the results?

No

Are sufficient details of methods and analysis provided to allow replication by others?

No

Reviewer Expertise:

Epidemiology and biostatistics

I confirm that I have read this submission and believe that I have an appropriate level of expertise to state that I do not consider it to be of an acceptable scientific standard, for reasons outlined above.

# Reviewer report: Reviewer response for version 2

**DOI:** 10.5256/f1000research.167506.r286910

**Published:** 2024-09-03

**Authors:** Said Moselhy

**Affiliations:** 1 Ain Shams University, Cairo, Egypt

The authors responded to comments. It is recommended for acceptance

Is the work clearly and accurately presented and does it cite the current literature?

No

If applicable, is the statistical analysis and its interpretation appropriate?

No

Are all the source data underlying the results available to ensure full reproducibility?

No

Is the study design appropriate and is the work technically sound?

No

Are the conclusions drawn adequately supported by the results?

No

Are sufficient details of methods and analysis provided to allow replication by others?

No

Reviewer Expertise:

Biochemistry

I confirm that I have read this submission and believe that I have an appropriate level of expertise to confirm that it is of an acceptable scientific standard.

# Reviewer report: Reviewer response for version 1

**DOI:** 10.5256/f1000research.153625.r241777

**Published:** 2024-05-14

**Authors:** Said Moselhy

**Affiliations:** 1 Ain Shams University, Cairo, Egypt

Dear editor

 - Subjects groups, sampling, exclusion and inclusion criteria , parameters, not clear.

 - What is the role of CYP450 for detoxification.

 -  GFR and clearance should be done.

 -  What about antioxidant activity?

 - How doses of exposure determined

Is the work clearly and accurately presented and does it cite the current literature?

No

If applicable, is the statistical analysis and its interpretation appropriate?

No

Are all the source data underlying the results available to ensure full reproducibility?

No

Is the study design appropriate and is the work technically sound?

No

Are the conclusions drawn adequately supported by the results?

No

Are sufficient details of methods and analysis provided to allow replication by others?

No

Reviewer Expertise:

Biochemistry

I confirm that I have read this submission and believe that I have an appropriate level of expertise to state that I do not consider it to be of an acceptable scientific standard, for reasons outlined above.

# Response

**Published:** 2024-06-03

**Authors:** Abdul Rohim Tualeka

**Affiliations:** Public Health, Universitas Airlangga, Surabaya, East Java, Indonesia

GFR and clearance should be done --- However, in this study, GFR and clearance measurements were not performed.
